# Evaluation of the Skin-Sensitizing Potential of Brazilian Green Propolis

**DOI:** 10.3390/ijms222413538

**Published:** 2021-12-17

**Authors:** Erina Shiraishi, Keishi Ishida, Daisuke Matsumaru, Akiko Ido, Youhei Hiromori, Hisamitsu Nagase, Tsuyoshi Nakanishi

**Affiliations:** 1 Laboratory of Hygienic Chemistry and Molecular Toxicology, Gifu Pharmaceutical University, 1-25-4 Daigaku-nishi, Gifu 501-1196, Gifu, Japan; 126033@gifu-pu.ac.jp (E.S.); ishida@gifu-pu.ac.jp (K.I.); matsumaru-da@gifu-pu.ac.jp (D.M.); aido@u-gifu-ms.ac.jp (A.I.); hiroy@suzuka-u.ac.jp (Y.H.); hnagase@u-gifu-ms.ac.jp (H.N.); 2 Japan Society for the Promotion of Science, 5-3-1 Kojimachi, Chiyoda-ku, Tokyo 102-0083, Japan; 3 Faculty of Pharmaceutical Sciences, Gifu University of Medical Science, 4-3-3 Nijigaoka, Kani 509-0293, Gifu, Japan; 4 Faculty of Pharmaceutical Sciences, Suzuka University of Medical Science 3500-3, Minamitamagaki, Suzuka 513-8670, Mie, Japan

**Keywords:** propolis, skin sensitization, contact dermatitis, human cell line activation test (h-CALT), local lymph node assay (LLNA), THP-1 cells, DC2.4 cells

## Abstract

Propolis is a resinous mixture produced by bees from their secretions and plant material, so its composition varies depending on its botanical origin. Propolis has several beneficial bioactivities, but its skin sensitization properties have long been suspected. Nevertheless, the skin sensitization potency of Brazilian green propolis (BGP) has not been scientifically evaluated. Here, we used scientifically reliable tests to evaluate it. In vitro antigenicity test based on the human cell line activation test (OECD TG 442E) was performed by measuring the expression of CD54 and CD86, which are indicators of the antigenicity of test substances, on THP-1 and DC2.4 cells. BGP did not affect the expression of either marker on THP-1 cells, but upregulated the expression of CD86 on DC2.4 cells, suggesting that BGP may be a skin sensitizer. Then, we performed local lymph node assay (LLNA, OECD TG 429) as a definitive in vivo test. LLNA showed that 1.70% BGP primed skin sensitization and is a “moderate sensitizer”. Our results indicate scientific proof of the validity of arbitrary concentrations (1–2%), which have been used empirically, and provide the first scientific information on the safe use of BGP.

## 1. Introduction

Propolis is a natural resinous mixture produced by honeybees from substances collected from parts of plants, such as buds, and exudates [1]. When bees chew on such materials, salivary enzymes are mixed with wax to produce propolis. In general, propolis is composed of 50% (*w*/*v*) resin and vegetable balsam, 30% (*w*/*v*) wax, 10% (*v*/*v*) essential and aromatic oils, 5% (*w*/*v*) pollen, and 5% other substances [2]. However, the composition of propolis varies depending on the geographical and botanical origin; more than 300 different compounds have been identified in propolis worldwide [2,3,4,5]. Propolis is classified into four main types: European, Brazilian, Cuban, and Taiwanese [6]. The composition of Taiwanese propolis is similar to that in Europe, with the main components being flavonoids, aromatic acids, and their esters [7]. Cuban propolis contains polyprenylated benzophenones [6]. Brazilian propolis (Baccharis type) contains less flavonoids and is richer in the cinnamic acid derivatives, such as Artepillin C than European propolis [8].

Propolis has many beneficial biological activities such as antibacterial, antiviral, immunomodulatory, anticancer, anti-inflammatory, and hepatoprotective properties [6,9,10,11,12,13,14]. Because of these benefits, it is widely used as a traditional medicine in many countries, especially in the Balkan states, the former USSR, Germany, and Austria. It is available in various forms such as food supplements, biopharmaceuticals, external preparations, shampoos, toothpastes, and cosmetics [9,15]. In spite of the benefits, propolis has long been suspected to have adverse effects. In particular, contact allergy is not rare and can be quite severe. For example, according to the European Surveillance System on Contact Allergies (ESSCA), positive (allergic) reactions to propolis patch tests were observed in 2.96% among 21,807 patients in 2015–2018 [16]. In the guinea pig maximization test, European propolis has been determined to be a skin sensitizer [17], and subsequently, some caffeic acid esters were identified as major substances in European propolis that induce skin sensitization [18,19]. These caffeic acid esters were also confirmed to cause skin sensitivity in clinical studies [18,19].

Recently, the incidence of contact dermatitis caused by propolis has increased in Europe [20] and North America [21], likely due to its use in cosmetic and pharmaceutical preparations. The premium Brazilian green propolis (BGP) produced in Southeast Brazil is traded worldwide and has been actively distributed in recent years because of its reliable quality [22]. Similar to European propolis, BGP has also been suspected to induce contact dermatitis. Nevertheless, BGP is widely used without evaluating its sensitizing properties. Currently, BGP is used in cosmetics, etc., at an arbitrary concentration (1–2%) [23] that is empirically considered to be safe; this assumption is not based on the results of scientifically reliable tests such as the skin sensitization test. BGP is composed mainly of cinnamic acid derivatives and does not contain caffeic acid esters, which are allergens in European propolis [8]. Therefore, if BGP has skin sensitization effects, it is necessary to characterize its action and determine the appropriate dosage by a scientifically reliable test.

In general, the potential of skin sensitization by chemicals is evaluated by the skin sensitization test, a globally recognized test method officially adopted in the Organisation for Economic Co-operation and Development Test Guidelines (OECD TG). In this test, the adverse outcome pathway leading to contact dermatitis after exposure to an allergen is described as a series of key events (KE): binding of chemical allergen to a protein as the molecular initiating event (KE1), keratinocyte activation (KE2), dendritic cell (DC) activation (KE3), and T cell activation and proliferation (KE4). Here, we evaluated the antigenicity of BGP corresponding to KE3 by the human cell line activation test (h-CLAT, OECD TG 442E) using THP-1 cells and a modified method using DC2.4 cells. We also evaluated the sensitization caused by BGP (corresponding to KE4) by local lymph node assay (LLNA, OECD TG 429). On the basis of these results, we discuss skin sensitization by BGP and its safe use.

## 2. Results and Discussion

### 2.1. Sensitization Potency of BGP in h-CLAT

The h-CLAT procedure stipulated in OECD TG 442E consists of two steps, both using THP-1 cells. In brief, the first step is dose-finding assay, and the second (main) step is the measurement of CD54 and CD86 expression on the cell surface. Dose-finding assay is conducted to determine the 75% cell viability (CV75) values of test substances; these values are then used to determine the optimal concentrations of the test substances in the main in vitro antigenicity test. In the main analysis, upregulation of the expression of CD54 and CD86, which are indicators of the antigenicity of test substances, is measured by flow cytometry after the treatment of cells with test substances. Prior to the routine use of the h-CLAT, the OECD guidelines recommend demonstrating technical proficiency by using any of the 10 proficiency substances. In accordance with the guidelines, we evaluated the sensitization potency of 2,4-dinitrochlorobenzene (DNCB), one of the recommended proficiency substances, before evaluating BGP. As expected, DNCB was positive for skin sensitization, indicating that we correctly conducted the h-CLAT procedure (Figure S1).

**Figure 1:**
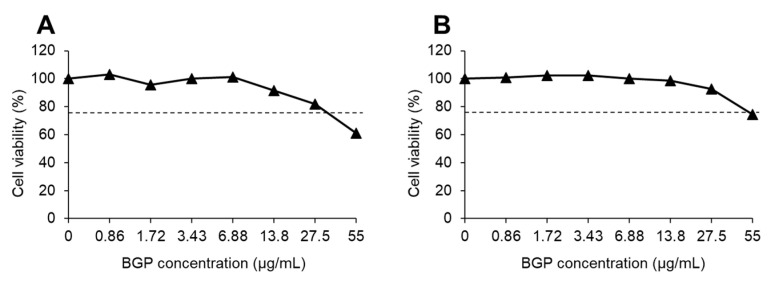
Viability of THP-1 cells treated with Brazilian green propolis (BGP). First experiment (**A**) and second experiment (**B**) were conducted independently, and data at each concentration were obtained from a single culture. Dashed lines indicate 75% cell viability.

**Figure 2:**
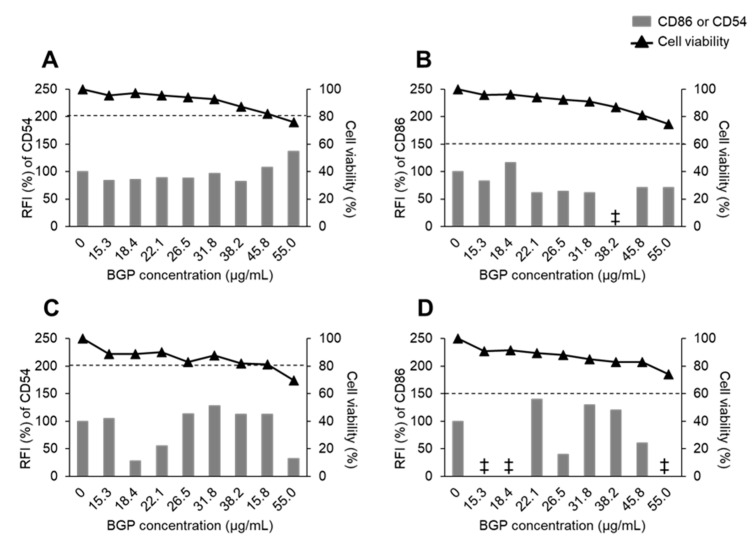
Viability of and expression of CD54 and CD86 on the cell surface of THP-1 cells treated with Brazilian green propolis (BGP). The expression of CD54 (**A**,**C**) and CD86 (**B**,**D**) was measured by flow cytometry, and the relative fluorescence intensity (RFI) values were calculated from the data. First experiment (**A**,**B**) and second experiment (**C**,**D**) were conducted independently, and data at each concentration were obtained from a single culture. Dashed lines indicate RFI values of 200% (CD54) or 150% (CD86). ‡: RFI < 0.

Then, we evaluated the sensitization potency of BGP by the h-CLAT procedure. In dose-finding assay, the CV75 values of BGP were calculated as 34.59 µg/mL in the first trial (Figure 1A) and 53.75 µg/mL in the second trial (Figure 1B), with an average of 44.17 µg/mL. The 1.2 × CV75 value was calculated from the CV75 value (44.17 µg/mL) to 53 µg/mL, which is almost the same as the maximum concentration of 55% BGP ethanol extract used in the current study. Therefore, we set BGP 55 µg/mL as the maximum dose, and eight doses (15.3, 18.4, 22.1, 26.5, 31.8, 38.2, 45.8, 55.0 μg/mL) were determined at a ratio of 1.2. Treatment with BGP did not alter CD54 or CD86 expression in the first trial (Figure 2A,B) or in the second trial (Figure 2C,D). These results of the h-CLAT suggest that BGP is a non-sensitizer.

### 2.2. Sensitization Potency of BGP in a Modified In Vitro Test Using DC2.4 Cells

The use of THP-1 cells in the h-CLAT may lead to false-negative results for some positive substances, such as α-hexyl cinnamaldehyde (HCA) [24]. When the skin is exposed to a chemical allergen, the chemical is recognized by Langerhans cells, a type of DC, which are the principal “professional” antigen-presenting cells in the epidermis and play a key role in the development of allergic contact dermatitis. Activation of DCs by chemical allergens corresponds to KE3. THP-1 is a monocyte cell line that can differentiate into DCs under certain conditions [25], but not under normal culture conditions, as indicated in the guidelines. This may be one of the causes of false negatives in the h-CLAT. To solve this problem, we previously proposed a test method using DC2.4 cells, a line of immature murine DCs, instead of THP-1 cells [26]. This cell line has been used as a DC model in immunological research [27,28,29,30,31]. Unlike THP-1 cells, DC2.4 cells respond positively to HCA [26], suggesting that the evaluation system using DC2.4 cells is more sensitive than that using THP-1 cells.

**Figure 3:**
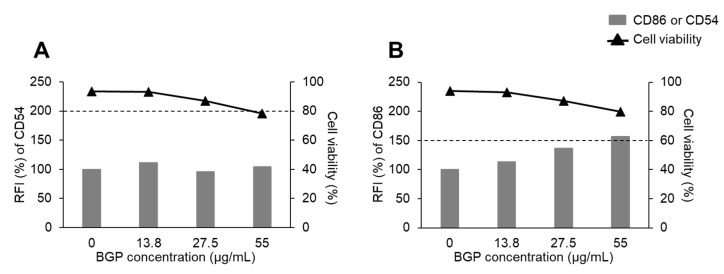
Viability of and expression of CD54 and CD86 on the cell surface of DC2.4 cells treated with Brazilian green propolis (BGP). The expression of CD54 (**A**) and CD86 (**B**) was measured by flow cytometry, and the relative fluorescence intensity (RFI) values were calculated. The data at each concentration were obtained from a single culture. Dashed lines indicate RFI values of 200% (CD54) or 150% (CD86).

When we tested DC2.4 cells, the expression level of CD54 was not noticeably changed by BGP stimulation (Figure 3A). On the other hand, the expression level of CD86 was increased in a dose-dependent manner. Moreover, the highest RFI value of CD86 was 156.73% (at 55 μg/mL BGP), which exceeded the positive threshold of 150% (Figure 3B). The result suggested that BGP may be a skin sensitizer. The difference in the results between ordinary the h-CLAT and the protocol using DC2.4 cells may be explained by sensitivity to antigen-induced activation of THP-1 cells and DC2.4 cells. That is, the high activation capacity of DC2.4 cells to antigens may indicate that they have more characteristics of dendritic cells compared to THP-1 cells, and this capacity resulted in the higher detection sensitivity of DC2.4 cells.

### 2.3. Evaluation of Skin Sensitization Potency of BGP by LLNA

To further characterize the sensitization potency of BGP, we next conducted LLNA, an in vivo sensitization test. Among animal tests, LLNA is considered the “gold standard” in estimating the allergenic potency of a chemical and is currently the first-choice method to assess sensitization potency [32]. LLNA can detect the activation and proliferation of T cells (corresponding to KE4) by measuring the incorporation of [^3^H]-thymidine during cell proliferation.

In the guidelines, young adult female CBA/Ca or CBA/J mice are the most highly recommended, but other strains or males can be used if sufficient data are available to demonstrate that there are no significant strain- or gender-specific differences in LLNA responses. The reactivity of BALB/c female mice in LLNA equals those of the CBA/Ca or CBA/J strains [33]. It is also recommended that a sensitization assessment is performed using positive control substances with known sensitizing strengths to ensure that this test is being performed properly. Therefore, we used the BALB/c strain and checked the competency of our laboratory and sensitivity of the assay in our experimental conditions by evaluating a positive control substance, HCA, before evaluating BGP. Our results indicated that the EC3 value of HCA was within the range of reference value in LLNA guidelines (Figure S2), suggesting that LLNA was performed properly (see Section 3.7 for the calculation of SI and EC3).

**Figure 4:**
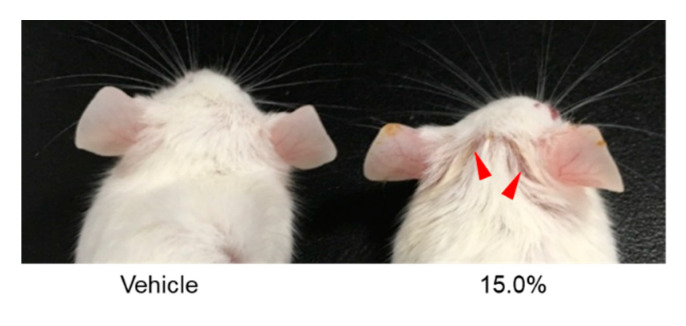
Ear inflammation induced by Brazilian green propolis (BGP). Vehicle or 15.0% (*w*/*v*) BGP was applied to the back of the ears of mice for 3 consecutive days for LLNA. In LLNA, all mice treated with 15.0% (*w*/*v*) BGP showed obvious ear inflammation at day 5 from the first exposure. The photos show the typical ears of control mouse (Vehicle; left photo) and inflamed mouse (15.0% BGP; right photo), respectively. Red arrowheads indicate the inflammatory response.

**Figure 5:**
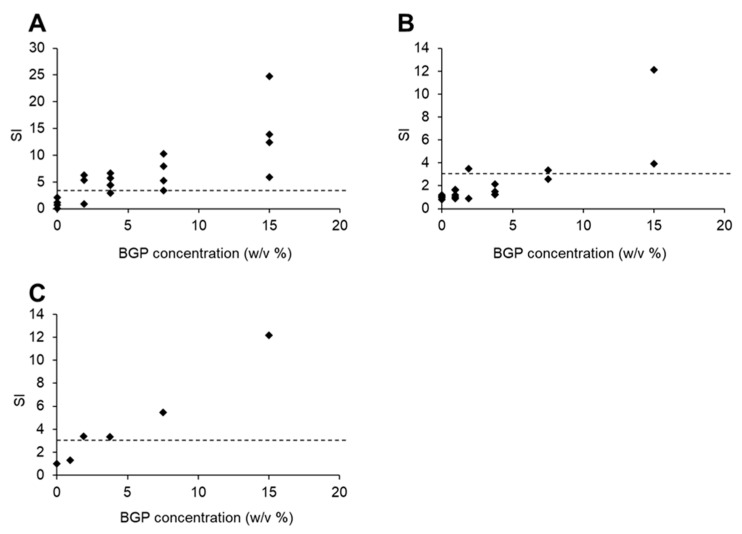
Evaluation of contact sensitization response to Brazilian green propolis (BGP) by LLNA. The stimulation index (SI) values of each mouse were calculated from DPM values (incorporation of [^3^H]-thymidine in auricular lymph node cells). (**A**) First experiment (*n* = 3–5); (**B**) second experiment (*n* = 2–5). (**C**) Mean SI values calculated from the data shown in (**A**,**B**). Dashed lines indicate SI = 3.

Then, we evaluated the sensitization potency of BGP by LLNA using the BALB/c strain. Mice treated with 15.0% (*w*/*v*) BGP for 3 consecutive days showed obvious ear inflammation on day 5, unlike mice treated with vehicle alone (Figure 4). The results of the first LLNA performed with BGP (Figure 5A) were broadly suggestive of a dose response. The mean DPM values of draining lymph node cells were 1377.2 and 4685.4 when mice were treated with 1.88% and 15.0% (*w*/*v*) BGP, respectively, and 328.7 in vehicle-treated controls (Table 1). However, contrary to expectations, the SI value exceeded 3 at the minimum concentration of 1.88%, and the EC3 value could not be calculated (Figure 5A). Therefore, a second LLNA was performed using a lower concentration of BGP to obtain EC3 values and to confirm reproducibility. Similar to the first experiment, the mean DPM values of draining lymph node cells in the 15.0% (*w*/*v*) BGP group were markedly higher than those in vehicle-treated controls (Figure 5B, Table 1). The mean DPM value for the 0.94% (*w*/*v*) BGP group was 422.8, close to 329.8 in vehicle-treated controls (Table 1). Finally, we compiled in Figure 5C the data from Figure 5A,B to obtain a dose response for BGP. The EC3 value of BGP calculated from the averaged data plotted as a full dose–response curve (Figure 5C) was 1.70%, indicating that BGP is a “moderate” skin sensitizer.

In the current study, we conducted two guideline tests and one modified test to evaluate the skin sensitization potency of BGP. BGP was judged negative in the ordinary h-CLAT, but positive in the modified in vitro test using DC2.4 cells and in LLNA. Because LLNA is widely accepted in the skin sensitization field as a standalone test, we finally concluded that BGP is a skin sensitizer and considered that the result of the ordinary h-CLAT was false negative. Among non-animal test methods that are accepted in the OECD test guidelines, the h-CLAT is technically applicable to the testing of multi-constituent substances and mixtures such as botanical extracts [34]. However, highly lipophilic test substances can lead to incorrect results in the h-CLAT [35]. For example, HCA is a highly lipophilic compound used as a positive control in LLNA, but it is negative in the h-CLAT [24]. Therefore, sensitizer substances present in BGP may also be highly lipophilic, leading to an incorrect conclusion. In contrast to THP-1 cells, DC2.4 cells were able to detect the skin sensitization potency of BGP. We previously reported that DC2.4 cells detect the skin sensitization potency of HCA, which cannot be detected by THP-1 cells [26]. Our current results also suggest that an in vitro test using DC2.4 cells can produce results closer to the results of LLNA than that using THP-1 cells.

Skin sensitization by European propolis has been detected in the guinea pig maximization test [17] and clinical patch test [16], which are more reliable than LLNA, and major skin sensitizers have been identified. 1,1-Dimethylallyl caffeic acid ester (LB-1) was the first sensitizer identified in European propolis [18]. Among other components, some caffeic acids such as benzyl caffeate and 3-methyl-2-butyl caffeate are strong skin sensitizers, whereas cinnamic acid and benzoic acid are weak sensitizers [18]. However, no skin sensitization by flavonoids such as kaempferol has been detected [19]. Given that the BGP used in this study consists mainly of cinnamic acid derivatives and flavonoids (Table 2), the skin sensitization property of BGP may be derived from a cinnamic acid derivative. Further studies are needed to identify the BGP components that cause skin sensitization. While BGP is used in a variety of products such as cosmetics, its safe concentration has not yet been scientifically confirmed.

## 3. Materials and Methods

### 3.1. Test Substances

The BGP (Minas Gerais State, Brazil) used in the current study originates mainly from *Baccharis dracunculifolia*. The Baccharis propolis extracted with 95% (*v*/*v*) ethanol/water mixture was kindly provided by API (Gifu, Japan) and used as a BGP solution. The content of the major components of solid BGP (55% solid content) used in this study are shown in Table 2; the data were kindly provided by API. The BGP solution was dissolved in dimethyl sulfoxide (DMSO; Fujifilm Wako, Osaka, Japan) for in vitro tests and in acetone/olive oil (4:1 *v*/*v*, AOO) for LLNA.

### 3.2. Cell Cultures

THP-1 cells (NIHS (JCRB) No. JCRB0112.1) used in h-CLAT were purchased from the JCRB Cell Bank. Available online: https://cellbank.nibiohn.go.jp/english/ (accessed on 13 December 2021). DC2.4 cells, a murine DC line [36], were kindly provided by Dr. Kenneth Rock (University of Massachusetts Medical Center, Worcester, MA, USA). Both cell lines were grown in RPMI1640 medium (Nacalai Tesque, Kyoto, Japan) supplemented with 10% heat-inactivated fetal bovine serum (Gibco by Life Technologies), 0.1 mM modified Eagle medium non-essential amino acid solution (Nacalai Tesque), and 0.05 mM 2-mercaptoethanol (Nacalai Tesque). The cells were maintained at 37 °C in a humidified incubator under 5% CO_2_. They were grown to confluence and passaged every 2–3 days. Thereafter, for the dose-finding assay and in vitro antigenicity test, THP-1 cells (1.0 × 10^6^ cells/well) were seeded in 24-well plates (Corning Inc., Corning, NY, USA) and treated with either various concentrations of BGP in 0.2% DMSO/0.02% ethanol or vehicle alone simultaneously for 24 h. DC2.4 cells (1.5 × 10^5^ cells/well) were seeded in 24-well plates (Corning), precultured at 37 °C for 24 h and then treated with either various concentrations of BGP or vehicle for 24 h. In control experiments, vehicle treatment had no significant effect in either test.

### 3.3. Animals

Specific pathogen–free female BALB/cCrSlc mice of 7–8 weeks of age were obtained from Japan SLC, Inc. (Shizuoka, Japan). Mice were housed in a room maintained at 23 ± 2 °C with 50% ± 10% humidity and a 12:12-h light–dark cycle (lights on from 8:00 a.m. to 8:00 p.m.) and fed a standard chow diet (CE-2; CLEA, Tokyo, Japan). Food and water were provided ad libitum. All animal care and handling procedures were approved by the Institutional Animal Care and Use Committee of Gifu Pharmaceutical University. All efforts were made to minimize both the number of animals used and the pain and distress they experienced.

### 3.4. Detection of Cell Viability

Dose-finding assay was conducted mainly in accordance with OECD TG No. 442E (h-CLAT) to determine the optimal concentration for the in vitro antigenicity test. Cells were treated with seven 2-fold serial dilutions of BGP or with vehicle alone, harvested, and then stained with 7-amino-actinomycin D (7-AAD) Viability Staining Solution (Thermo Fisher Scientific, Waltham, MA, USA). The cells were analyzed using FACSVerse (BD Biosciences, Franklin Lakes, NJ, USA). The CV75 value was calculated by log-linear interpolation as follows:

log CV75 = [(75 − C) × log D − (75 − A) × log B]/(A − C)

where A is the minimum cell viability >75% in the test group, C is the maximum cell viability <75% in the test group, and B and D are the concentrations at which the cell viabilities are A and C, respectively. A total of eight doses were set on the basis of CV75: 1/1.2^6^ × CV75, 1/1.2^5^ × CV75, 1/1.2^4^ × CV75, 1/1.2^3^ × CV75, 1/1.2^2^ × CV75, 1/1.2 × CV75, CV75, and 1.2 × CV75.

### 3.5. In Vitro Antigenicity Test

In vitro antigenicity test was conducted mainly in accordance with OECD TG No. 442E (h-CLAT). THP-1 cells were treated with eight 1.2-fold serial dilutions of BGP starting at 1.2 × CV75 or with vehicle alone. The cells were harvested and then pretreated with Human BD Fc block (BD Biosciences) to avoid nonspecific binding of primary antibodies. The cells were stained with biotinylated antibody against human CD54 or human CD86 (both from Thermo Fisher Scientific) for 30 min. After washing, the cells were stained with FITC–streptavidin (BD Biosciences) for another 30 min. After washing, the cells were stained with 7-AAD to gate out dead cells.

DC2.4 cells were treated with three 2-fold serial dilutions of BGP starting at 1.2 × CV75 or with vehicle alone. Antibodies specific to the mouse surface molecules (Mouse BD Fc Block; biotinylated anti-mouse CD54 antibody; biotinylated anti-mouse CD86 antibody; all from BD Biosciences) were used for staining instead of antibodies against human surface molecules. Expression of CD54 and CD86 was analyzed by FACSVerse.

For each cell line, live cells in the collected 30,000 cells were analyzed. The raw data represented by geometric mean fluorescence intensity (MFI) were used to calculate relative fluorescence intensity (RFI), an indicator of CD54 and CD86 expression, as follows:

RFI (%) = 100 × (MFI of chemical-treated cells)/(MFI of vehicle-only cells)

If the RFI was equal to or greater than 200% (CD54) or 150% (CD86) at any dose in at least two independent experiments, the test chemical was considered a sensitizer; otherwise, it was considered a non-sensitizer.

### 3.6. LLNA

LLNA was conducted mainly in accordance with OECD TG No. 429. Groups of BALB/c female mice (8 weeks of age) were treated topically on the dorsum of ears with 25 µL of BGP at 4 concentrations in 2-fold serial dilutions or with an equal volume of vehicle alone (AOO). Treatment was performed daily for 3 consecutive days (days 0, 1, and 2). On day 5, all mice were injected through the tail vein with 0.25 mL [methyl-^3^H]-thymidine (20 μCi; American Radiolabeled Chemicals, St. Louis, MO, USA). After 5 h, mice were euthanized by a lethal overdose of isoflurane, the auricular lymph nodes, as the draining lymph nodes, were excised and lymph node cells were collected individually. Each lymph node cell suspension was washed with phosphate-buffered saline, pelleted by centrifugation, and then resuspended in 5 mL of 5% (*v*/*v*) trichloroacetic acid (TCA) (Fujifilm Wako) and precipitated at 4 °C for 18 h. After centrifugation of the cell solution, pellets were resuspended in 1 mL of 5% TCA, and each sample was mixed with 4 mL of scintillation cocktail Clear-sol I (Nacalai Tesque). Incorporation of [methyl-^3^H]-thymidine was measured by β-scintillation counting and expressed as disintegrations per minute (DPM) per mouse.

### 3.7. Estimation of Skin Sensitization Potency from LLNA Results

In LLNA, the evaluation of skin sensitization potency is based on stimulation index (SI) values. A chemical is classified as a skin sensitizer when its proliferative activity is at least 3 times that of vehicle. The SI value was calculated using the mean DPM value for each dose group as the numerator and the mean DPM value for the vehicle control as the denominator. A test material that causes an at least 3-fold increase in proliferation at one or more concentrations is considered to be positive in the LLNA test. As a measure of sensitizing potency, we used EC3 values % (the estimated concentration that induces a three-fold SI as compared to the respective vehicle) calculated by linear interpolation according to the equation:

EC3 = *c* + [(3 − *d*)/(*b* − *d*)](*a* − *c*)

where the data points lying immediately above and below the SI value of 3 on the LLNA dose–response plot have the co-ordinates (*a*, *b*) and (*c*, *d*), respectively [37]. The EC3 values were used to subcategorize chemicals according to their potency: extreme (0% < EC3 < 0.1%), strong (0.1% ≤ EC3 < 1.0%), moderate (1.0% ≤ EC3 < 10%), or weak (10% ≤ EC3 ≤ 100%) [38]. The % here indicates either *v*/*v* or *w*/*v*.

## 4. Conclusions

In the current study, we identified the minimum concentration that induces skin sensitization as 1.70%. Our results provide useful scientific information about BGP safety in everyday use, and scientifically proves the appropriateness of the arbitrary concentration (1–2%) that has been used empirically [23].

## Figures and Tables

**Table 1:** Detailed data of local lymph node assay for Brazilian green propolis (BGP).

Experiment	Concentration % (*w*/*v*)	DPM ^a^ (Mean or Mean ± 1 S.D.)	Number of Animals Used
Exp. 1	0.00	328.7 ± 276.5	4
	1.88	1377.2 ± 939.8	3
	3.75	1587.2 ± 467.5	5
	7.50	2208.0 ± 983.4	4
	15.00	4685.4 ± 2574.1	4
Exp. 2	0.00	329.8 ± 55.3	4
	0.94	422.8 ± 113.1	5
	1.88	720.9	2
	3.75	501.3 ± 143.7	4
	7.50	983.8	2
	15.00	2644.9	2

^a^ DPM, disintegrations per minute.

**Table 2:** Components and their contents in Brazilian green propolis (BGP) used in the current study.

Component Classification	Compound Name	Structure	Content (mg/g) ^a^
Cinnamic acid derivatives	Artepillin C	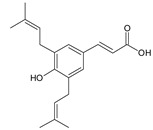	163.87
	Baccharin	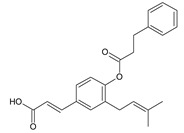	47.31
	Drupanin	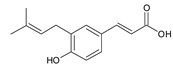	19.89
	*p*-Coumaric acid	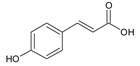	21.95
Flavonoids	Kaempferide	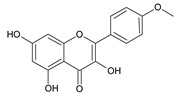	27.79
	Dihydrokaempferide	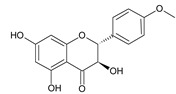	16.41
	Betuletol	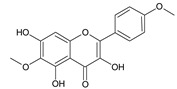	16.44
	Isosakuranetin	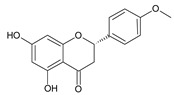	4.83
	Kaempferol	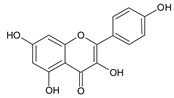	2.05

^a^ The value of content indicates weight (mg) in 1 gram of solid BGP.

## Data Availability

Not applicable.

## Supplementary Materials

The following are available online at https://www.mdpi.com/article/10.3390/ijms222413538/s1.

## References

[1] Wagh V.D. (2013). Propolis: A wonder bees product and its pharmacological potentials. Adv. Pharmacol. Sci..

[2] Burdock G.A. (1998). Review of the biological properties and toxicity of bee propolis (propolis). Food Chem. Toxicol..

[3] Huang S., Zhang C.P., Wang K., Li G.Q., Hu F.L. (2014). Recent advances in the chemical composition of propolis. Molecules.

[4] Kasiotis K.M., Anastasiadou P., Papadopoulos A., Machera K. (2017). Revisiting Greek Propolis: Chromatographic Analysis and Antioxidant Activity Study. PLoS ONE.

[5] Silva-Beltrán N.P., Balderrama-Carmona A.P., Umsza-Guez M.A., Souza Machado B.A. (2020). Antiviral effects of Brazilian green and red propolis extracts on Enterovirus surrogates. Environ. Sci. Pollut. Res. Int..

[6] Bankova V. (2005). Recent trends and important developments in propolis research. Evid.-Based Complement. Alternat. Med..

[7] Sforcin J.M., Bankova V. (2011). Propolis: Is there a potential for the development of new drugs?. J. Ethnopharmacol..

[8] Gardana C., Simonetti P. (2011). Evaluation of allergens in propolis by ultra-performance liquid chromatography/tandem mass spectrometry. Rapid Commun. Mass Spectrom..

[9] De Groot A.C. (2013). Propolis: A review of properties, applications, chemical composition, contact allergy, and other adverse effects. Dermatitis.

[10] Berretta A.A., Arruda C., Miguel F.G., Baptista N., Nascimento A.P., Marquele-Oliveira F., Hori J.I., Barud H., Damaso B., Ramos C. (2017). Functional Properties of Brazilian Propolis: From Chemical Composition until the Market.

[11] Talas Z.S., Gulhan M.F., Erdogan K., Orun I. (2014). Antioxidant effects of propolis on carp *Cyprinus carpio* exposed to arsenic: Biochemical and histopathologic findings. Dis. Aquat. Organ..

[12] Machado B.A.S., de Abreu Barreto G., Costa A.S., Costa S.S., Silva R.P.D., da Silva D.F., Brandão H.N., da Rocha J.L.C., Nunes S.B., Umsza-Guez M.A. (2015). Determination of Parameters for the Supercritical Extraction of Antioxidant Compounds from Green Propolis Using Carbon Dioxide and Ethanol as Co-Solvent. PLoS ONE.

[13] Devequi-Nunes D., Machado B.A.S., de Abreu Barreto G., Rebouças Silva J., da Silva D.F., da Rocha J.L.C., Brandão H.N., Borges V.M., Umsza-Guez M.A. (2018). Chemical characterization and biological activity of six different extracts of propolis through conventional methods and supercritical extraction. PLoS ONE.

[14] Badr G., Sayed E.A., Waly H., Hassan K.A.H., Mahmoud M.H., Selamoglu Z. (2019). The Therapeutic Mechanisms of Propolis Against CCl4-Mediated Liver Injury by Mediating Apoptosis of Activated Hepatic Stellate Cells and Improving the Hepatic Architecture through PI3K/AKT/mTOR, TGF-β/Smad2, Bcl2/BAX/P53 and iNOS Signaling Pathways. Cell. Physiol. Biochem. Int. J. Exp. Cell. Physiol. Biochem. Pharmacol..

[15] Walgrave S.E., Warshaw E.M., Glesne L.A. (2005). Allergic contact dermatitis from propolis. Dermatitis.

[16] Uter W., Bauer A., Belloni A.F., Bircher A.J., Brans R., Buhl T., Cooper S.M. (2021). Patch Test Results with the European Baseline Series and Additions Thereof in the ESSCA Network, 2015–2018. Contact Dermat..

[17] Petersen H.O. (1977). Hypersensitivity to propolis. Contact Dermat..

[18] Hausen B.M., Wollenweber E., Senff H., Post B. (1987). Propolis allergy. (II). The sensitizing properties of 1,1-dimethylallyl caffeic acid ester. Contact Dermat..

[19] Hausen B.M., Evers P., Stüwe H.-T., König W.A., W7ollenweber E. (1992). Propolis Allergy (IV). Studies with Further Sensitizers from Propolis and Constituents Common to Propolis, Poplar Buds and Balsam of Peru. Contact Dermat..

[20] Uter W., Gefeller O., Mahler V., Geier J. (2020). Trends and Current Spectrum of Contact Allergy in Central Europe: Results of the Information Network of Departments of Dermatology (IVDK) 2007–2018. Br. J. Dermatol..

[21] DeKoven J.G., Silverberg J.I., Warshaw E.M., Atwater A.R., Reeder M.J., Sasseville D., Taylor J.S., Zug K.A., Belsito D.V., Maibach H.I. (2021). North American Contact Dermatitis Group Patch Test Results: 2017–2018. Dermatitis.

[22] Yang C., Luo L., Zhang H., Yang X., Lv Y., Song H. (2010). Common aroma-active components of propolis from 23 regions of China. J. Sci. Food Agric..

[23] Bogdanov S. (2017). Propolis: Biological properties and medical applications. The Propolis Book.

[24] Nukada Y., Ashikaga T., Miyazawa M., Hirota M., Sakaguchi H., Sasa H., Nishiyama N. (2012). Prediction of skin sensitization potency of chemicals by human Cell Line Activation Test (h-CLAT) and an attempt at classifying skin sensitization potency. Toxicol. Vitr..

[25] Berges C., Naujokat C., Tinapp S., Wieczorek H., Hoeh A., Sadeghi M., Opelz G., Daniel V. (2005). A cell line model for the differentiation of human dendritic cells. Biochem. Biophys. Res. Commun..

[26] Shiraishi E., Ido A., Hiromori Y., Tanaka K., Kimura T., Nagase H., Nakanishi T. (2017). Utility of Murine Dendritic Cell Line DC2.4 for in Vitro Assay of Skin-Sensitization Potential. Fundam. Toxicol. Sci..

[27] Fang F., Wang Y., Li R., Zhao Y., Guo Y., Jiang M., Sun J., Ma Y., Ren Z., Tian Z. (2010). Transcription Factor E2F1 Suppresses Dendritic Cell Maturation. J. Immunol..

[28] Hayashi A., Wakita H., Yoshikawa T., Nakanishi T., Tsutsumi Y., Mayumi T., Mukai Y., Yoshioka Y., Okada N., Nakagawa S. (2007). A strategy for efficient cross-presentation of CTL-epitope peptides leading to enhanced induction of in vivo tumor immunity. J. Control. Release.

[29] Kato S., Koizumi K., Yamada M., Inujima A., Takeno N., Nakanishi T., Sakurai H., Nakagawa S., Saiki I. (2010). A phagocytotic inducer from herbal constituent, pentagalloylglucose enhances lipoplex-mediated gene transfection in dendritic cells. Biol. Pharm. Bull..

[30] Okada N., Tsujino M., Hagiwara Y., Tada A., Tamura Y., Mori K., Saito T., Nakagawa S., Mayumi T., Fujita T. (2001). Administration route-dependent vaccine efficiency of murine dendritic cells pulsed with antigens. Br. J. Cancer.

[31] Takekoshi T., Tada Y., Watanabe T., Sugaya M., Hoashi T., Komine M., Kawashima T., Shimizu T., Hau C.S., Asahina A. (2010). Identification of a Novel Marker for Dendritic Cell Maturation, Mouse Transmembrane Protein 123. J. Biol. Chem..

[32] Angers-Loustau A., Tosti L., Casati S. (2011). The Regulatory Use of the Local Lymph Node Assay for the Notification of New Chemicals in Europe. Regul. Toxicol. Pharmacol..

[33] Woolhiser M.R., Munson A.E., Meade B.J. (2000). Comparison of mouse strains using the local lymph node assay. Toxicology.

[34] Nishijo T., Miyazawa K., Saito Y., Otsubo H., Mizumachi H., Sakaguchi H. (2019). Sensitivity of KeratinoSens™ and H-CLAT for Detecting Minute Amounts of Sensitizers to Evaluate Botanical Extract. J. Toxicol. Sci..

[35] Takenouchi O., Miyazawa M., Saito K., Ashikaga T., Sakaguchi H. (2013). Predictive performance of the human Cell Line Activation Test (h-CLAT) for lipophilic chemicals with high octanol-water partition coefficients. J. Toxicol. Sci..

[36] Shen Z., Reznikoff G., Dranoff G., Rock K.L.G. (1997). Cloned Dendritic Cells Can Present Exogenous Antigens on Both MHC Class I and Class II Molecules. J. Immunol..

[37] Basketter D.A., Lea L.J., Dickens A., Briggs D., Pate I., Dearman R.J., Kimber I. (1999). A Comparison of Statistical Approaches to the Derivation of EC3 Values from Local Lymph Node Assay Dose Responses. J. Appl. Toxicol..

[38] Kimber I., Basketter D.A., Berthold K., Butler M., Garrigue J.L., Lea L., Newsome C., Roggeband R., Steiling W., Stropp G. (2001). FORUM Skin Sensitization Testing in Potency and Risk Assessment. Toxicol. Sci..

