# Supplementary material for: Evaluation of the Skin-Sensitizing Potential of Brazilian Green Propolis
Source: Int J Mol Sci. 2021 Dec 17;22(24):13538. doi: 10.3390/ijms222413538 (PMC8704603; doi:10.3390/ijms222413538)
Supplement: Supplementary file 1 [file ijms-22-13538-s001.zip › ijms-1487596-supplementary.pdf]

Supplemental information

Evaluation of the Skin-Sensitizing Potential of Brazilian Green Propolis

Erina Shiraishi [1,2], Keishi Ishida [1], Daisuke Matsumaru [1], Akiko Ido [1,3], Youhei Hiromori [1,4], Hisamitsu Nagase [1,3] and Tsuyoshi Nakanishi [1,*]

1 Laboratory of Hygienic Chemistry and Molecular Toxicology, Gifu Pharmaceutical University, 1-25-4 Daigaku-nishi, Gifu 501-1196, Gifu, Japan; 126033@gifu-pu.ac.jp (E.S.); ishida@gifu-pu.ac.jp (K.I.); matsumaru-da@gifu-pu.ac.jp (D.M.); aido@u-gifu-ms.ac.jp (A.I.); hiroy@suzuka-u.ac.jp (Y.H.); hnagase@u-gifu-ms.ac.jp (H.N.)

2 Research Fellow of the Japan Society for the Promotion of Science, 5-3-1 Kojimachi, Chiyoda-ku, Tokyo 102-0083, Japan

3 Faculty of Pharmaceutical Sciences, Gifu University of Medical Science, 4-3-3 Nijigaoka, Kani 509-0293, Gifu, Japan

4 Faculty of Pharmaceutical Sciences, Suzuka University of Medical Science 3500-3, Minamitamagaki, Suzuka 513-8670, Mie, Japan

* Correspondence: nakanishi@gifu-pu.ac.jp; Tel.: +81-58-230-8100; Fax: +81-58-230-8117

Supplementary notes

Proficiency substances

2,4-Dinitrochlorobenzene (DNCB; Fujifilm Wako, Osaka, Japan) was used as a positive control for *in vitro* antigenicity tests. α-hexyl cinnamaldehyde (HCA; Fujifilm Wako) was used as positive controls in local lymph node assay (LLNA, OECD TG 429). DNCB was dissolved in dimethyl sulfoxide (Fujifilm Wako). HCA was dissolved in acetone/olive oil (4:1 v/v, AOO).

Validation of the reliability of h-CLAT conducted in our laboratory using DNCB

Prior to the routine use of the human cell line activation test (h-CLAT, OECD TG 442E), the guidelines recommend to demonstrate technical proficiency using "proficiency substances". Therefore, we checked whether the sensitization by DNCB, a well-known positive control substance, could be correctly evaluated by h-CLAT in our laboratory. A dose-finding assay for DNCB was conducted twice, and the CV75 was calculated to be 4.00 μg/mL (Fig. S1A) and 1.71 μg/mL (Fig. S1B), respectively, and the average of 2.86 μg/mL was calculated as the mean CV75. In h-CLAT guideline, the reference range of CV75 for DNCB are set to 2.00–12.0 μg/mL, and its median is 7.00 μg/mL. Therefore, the average of the two values of 2.86 μg/mL and 7.00 μg/mL was calculated to be 4.93 μg/mL, and we defined it as the final CV75 value. The following eight doses were set on the basis of the final CV75: 0.89, 1.76, 2.11, 2.53, 3.03, 3.65, 4.38, and 5.25 μg/mL (Fig. S1C–F). Then, the main test—CD54 and CD86 expression measurement—was conducted. In the first trial, the highest relative fluorescence intensity (RFI) values of CD54 and CD86 calculated from the equation described in Materials and methods section 2.5 were 661.4% and 240.0%, respectively, which exceeded the respective thresholds of 200% and 150% (dashed lines in Fig. S1C, D). In the second trial, the highest RFI values of CD54 and CD86 were 1068% and 1224%, respectively, which also exceeded the thresholds (Fig. S1E, F). In h-CLAT, the conclusion should be based on at least two independent tests. If the RFI value is equal to or greater than 200% (CD54) or 150% (CD86) at any dose in at least two independent experiments, the test chemical is considered a sensitizer; otherwise, it is considered a non-sensitizer. Therefore, we determined DNCB to be a sensitizer, and this result confirms that our test was performed correctly.

Validation of the reliability of LLNA conducted in our laboratory using HCA

In accordance with the OECD TG 429, the reliability of LLNA in our laboratory was validated by using HCA in BALB/c mice. Disintegrations per minute (DPM) represent ^{3}H-thymidine incorporation into the draining lymph node cells of mice after exposure to vehicle or various concentrations of HCA. The positive control substance produces a positive response (stimulation index [SI] ≥ 3). The DPM values were increased from 357.11 (vehicle) to 701.78 and 2030.18 after treatment with 5.0% and 15.0% (v/v) of HCA, respectively. (Fig. S2A). From this data, we also calculated the SI values (Fig. S2B) and concentrations at which the SI value was 3, called EC3. The EC3 value of HCA was 8.98% and which was within the range of reference values (4.8%–19.5%) in LLNA guidelines (Fig. S2B), indicating that LLNA was performed appropriately in our laboratory.

Supporting Figures

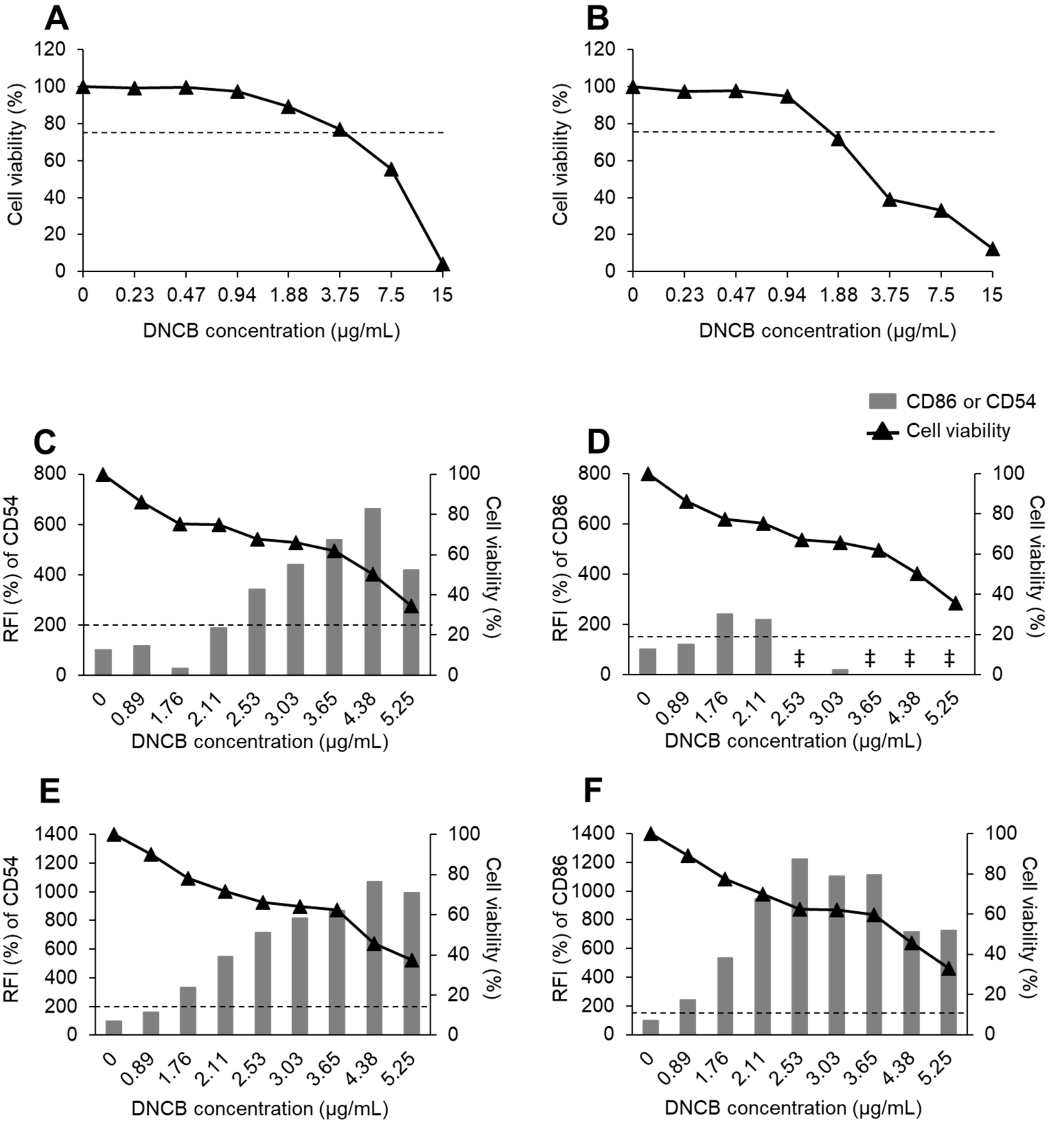

Figure S1. Cell viability and expression of CD54 and CD86 on THP-1 cells treated with DNCB. Cells were treated with DNCB at various concentrations for 24 h. (**A** and **B**) Dose-finding assays were performed by staining dead cells with 7-amino-actinomycin D and detecting them by flow cytometry. First experiment (**A**) and second experiment (**B**) were conducted independently, and data at each concentration were obtained from a single culture. Dashed lines indicate 75% cell viability. (**C**–**F**) The expression of CD54 (**C** and **E**) and CD86 (**D** and **F**) was measured by flow cytometry, and the relative fluorescence intensity (RFI) values were calculated from the data. First experiment (**C** and **D**) and second experiment (**E** and **F**) were conducted independently, and data at each concentration were obtained from a single culture. Dashed lines indicate RFI values of 200% (CD54) or 150% (CD86). ‡: RFI < 0.

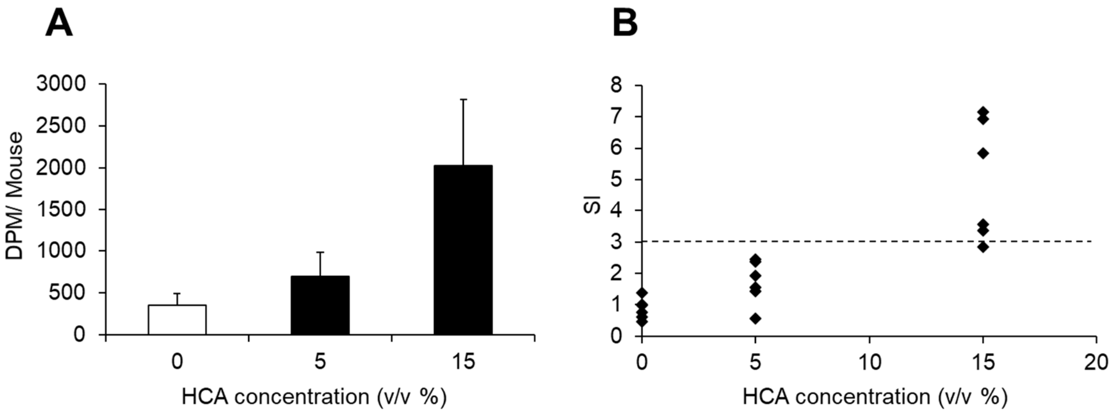

Figure S2. Evaluation of contact sensitization response for HCA by LLNA. Mice were treated with vehicle alone, 5.0% HCA or 15.0% HCA (each group n = 6) applied to both ears daily for 3 consecutive days. (**A**) Incorporation of [^{3}H]-thymidine into auricular lymph node cells was expressed as the mean DPM ± 1SD. (**B**) Stimulation index (SI) values were calculated from DPM values. Dashed lines indicate SI = 3.
